# Do laypersons need App-linked real-time feedback devices for effective resuscitation? – Results of a prospective, randomised simulation trial

**DOI:** 10.1016/j.resplu.2024.100631

**Published:** 2024-04-17

**Authors:** Sabine Wingen, Nele Großfeld, Niels-Benjamin Adams, Antonia Streit, Jan Stock, Bernd W. Böttiger, Wolfgang A. Wetsch

**Affiliations:** a University of Cologne, Medical Faculty and University Hospital Cologne, Department of Anaesthesiology and Intensive Care Medicine, Kerpener Str. 62, 50937 Cologne, Germany; b German Resuscitation Council, Prittwitzstraße 43, 89070 Ulm, Germany; c FOM University of Applied Sciences, Agrippinawerft 4, 50678 Cologne, Germany; d University of Applied Science Stralsund, Faculty of Electrical Engineering and Computer Science, Zur Schwedenschanze 15, 18435 Stralsund, Germany; e L2R GmbH, Cliev 4, 51515 Kürten, Germany; f University of Cologne, Medical Faculty, Albertus-Magnus-Platz, 50923 Cologne, Germany

**Keywords:** Basic Life Support (BLS), Cardiopulmonary resuscitation (CPR), Cardiac arrest, Real-time feedback, Training, Mobile applications, Mobile health, Smartphone

## Abstract

**Background:**

App-linked real-time feedback-devices for cardiopulmonary resuscitation (CPR) aim to improve laypersons’ resuscitation quality. Resuscitation guidelines recommend these technologies in training settings. This is the first study comparing resuscitation quality of all App-linked feedback-devices currently on market.

**Methods:**

A prospective randomised simulation study was performed. After standardised instructions, participants performed 2-minutes compression-only CPR on a manikin without feedback (baseline). Afterwards, participants performed 4 × 2 min CPR with four different feedback devices in randomised order (CorPatch® Trainer, CPRBAND AIO Training, SimCPR®ProTrainer, Relay Response™) (intervention). CPR metrics (chest compression depth (CD), chest compression rate (CR), percentage of correct CD/CR (%), correct hand position, correct chest recoil, and technical preparation-time) were assessed. Devices data were compared to the baseline group using Wilcoxon testing with IBM SPSS (primary outcome). Differences between devices were analysed with ANOVA testing (secondary outcome). Normally distributed data were described as mean ± standard deviation (SD) and non-normally distributed data as Median [Interquartile range (IQR). CPR self-confidence was measured by means of questionnaire before and after feedback devices’ use. Comparison was performed by students *t*-test.

**Results:**

Forty participants were involved. SimCPR®ProTrainer was the only device, which resulted in guideline-compliant chest compressions (Mean ± SD:5.37 ± 0.76) with improved chest compression depth (*p* < 0.001), and percentage of correct chest compression depth (*p* < 0.001) compared to unassisted CPR (baseline). CorPatch® Trainer as the only device with audio-visual recoil instructions resulted in improved chest recoil (Mean ± SD:72.25 ± 24.89) compared to baseline (Mean ± SD:49.00 ± 42.20; *p* < 0.01), while the other three devices resulted in significantly lower chest recoil rates (CPRBAND AIO Training: 37.03 ± 39.90; *p* < 0.01, SimCPR®ProTrainer: Mean ± SD:39.88 ± 36.50; *p* = 0.03, Relay Response™: Mean ± SD:36.88 ± 37.73; *p* = 0.02). CPR quality when using the different feedback devices differ in chest compression depth (*p* = 0.02), chest compression rate (*p* < 0.001), percentage of correct chest compression depth/rate (*p* = 0.03/*p* = 0.04), and technical preparation-time (*p* < 0.001). Feedback-devices’ use increased participant’s CPR self-confidence (*p* < 0.001).

**Conclusion:**

Although, CPR feedback devices show improved CPR performance in layperson in some metrics, none of the tested CPR feedback devices supported layperson in overall adequate CPR performance. More and better technical functionality is necessary, to fully utilise the potential of CPR feedback devices and to prevent a worsening of CPR performance when layperson use this technology.

## Introduction

Probability of survival after out-of-hospital cardiac arrest has been shown to increase if bystander begin cardiopulmonary resuscitation (CPR) immediately, and before emergency medical services arrive on scene.^1^, ^2^ Therefore, education and training in resuscitation are common in societies and subjects of worldwide public health campaigns.^4^, ^5^ The European Resuscitation Guidelines clearly promote the development and integration of CPR feedback technologies in the chain of survival and in Basic-Life-Support actions.^6^, ^7^ Based on this, a large number of mobile technologies like applications (apps) for smartphones and tablets in combination with mobile technical real-time feedback devices (short: App-linked CPR feedback-devices) has been developed during the last years.^7^

App-linked CPR feedback-devices are freely accessible in App stores, without the need of quality control. Evidence for usefulness of smart devices in CPR training could not be confirmed in the past.^8^ Nor have these technologies for resuscitation training and real incident support for medical staff shown clear benefit.^9^, ^10^, ^11^ As cardiac arrest is a life-threatening event, guideline-compliant content, sufficient usability, and adequate performance feedback is of high importance in these technologies.^12^

Since a huge number of resuscitation apps and CPR feedback-devices for laypersons exist on market and scientific knowledge about quality, usability, and guideline-conformity is still lacking,^13^ we performed the QualiApp research project. Aim of the study is to investigate the quality of App-linked CPR feedback-devices for resuscitation training currently available on market for the target group of laypersons. The primary study objective is to evaluate if all CPR metrics (chest compression depth 5–6 cm, chest compression rate 100–120/min, correct hand position, correct recoil) could be improved by using an App-linked real-time feedback device during a resuscitation session (two minutes) compared to unassisted CPR. Secondary study objectives are: How do investigated App-linked CPR feedback devices differ in their quality of CPR metrics and technical preparation time, and how does the use of these technologies affect laypersons’ self-confidence in their own CPR competences?

## Material and methods

### Trial design

Single-center, prospective, randomised simulation study using standardised CPR manikins. The Ethics Committee of the University of Cologne approved the study (No. 23-1078; 23-03-2023; Head: Prof. Dr. Raymond Voltz), which was conducted in accordance with the Declaration of Helsinki. The study was registered at the German Registry of Clinical Trials (ID: DRKS00031876).

### Participants

Healthy adult volunteers aged 18–49 years without any medical background were eligible to participate in the study. Pregnant, breastfeeding, persons with medical background (doctors, nurses, emergency medical personnel), previously ill persons (e.g. heart disease), and persons who, due to their language skills, cannot fully follow the instructions of the apps were excluded from participation. Participants were recruited at the Campus of the University Hospital of Cologne during the study period in May 2023 by the study team.

The study took place at the Skills Lab at the University Hospital of Cologne. A full-scale CPR-manikin (Resusci Anne Q CPR AW Torso Rechargeable model, product no.: 172-00160; Laerdal, Stavanger, Norway) was used. The model reproduces a human torso and its resistance during resuscitation by means of a linear spring in the centre of the manikin. For the study setting, the spring with medium resistance (45 kg) was used. Data were stored on the SimPad Plus. Data export was done using the Laerdal Session Viewer for Windows (Laerdal, Stavanger, Norway).

### Interventions

After giving written and informed consent, participants were accompanied into the simulation room by a study assistant and received standardised, detailed CPR instructions (compression-only) for laypersons with a duration of approximately ten minutes.^3^ CPR instructions were provided by reading a pre-defined text including relevant information about (i) correct hand position, (ii) how to perform chest compressions (depth and rate), and chest recoil. In parallel, all CPR steps were demonstrated on the resuscitation manikin that was used during the study afterwards. Participants were instructed to provide two minutes compression-only CPR without any technical support (baseline). Afterwards, participants performed 2 min compression-only CPR with four different CPR feedback devices in randomised order (intervention). The time between each CPR session was approximately three minutes in which participants receive a short break. The time slot allowed participants to take a short break and was used to save technical data, renew the study setting, and explain the next device. Before starting the next CPR session participants were always asked if they feel ready.

Before the study was started, a systematic search in Google Play Store and Apple App Store was performed in April 2023 (PROSPERO Registration No. CRD42023408007) to identify all available feedback devices for the target group of laypersons. The following four mobile applications with real-time feedback devices were identified: (1) SimCPR®ProTrainer (SIMCPR Medical BV, Netherlands), (2) CorPatch® Trainer (SmartResQ ApS, Denmark) (3) CPRBAND AIO Training (CREDO Co., LtD., Korea), and (4) Relay Response™ (XiMio Health, Inc., United States of America). More details of investigated apps are given in Table 1. None of these products is a medical device, as the intended use of all of them refers to the use in the training setting. For the study setting, a smartphone, a tablet, and a smartwatch were used. The smartphone was a Samsung Galaxy A23 5G (Samsung Electronics Co., Ltd., South Korea). The tablet used was a Samsung Galaxy Tab S6 Lite (Samsung Electronics Co., Ltd., South Korea). During the study, both were operated with the software version Android 13. The smartwatch used was the Samsung Galaxy 5 (Samsung Electronics Co., Ltd., South Korea; software version WearOS 3.5). Before using each device, participants received detailed and standardised instructions for use by a second study assistance. Afterward, participants were asked to prepare themselves with the technique and report when they were ready for CPR. The study assistants ended CPR after two minutes for all CPR attempts.

**Table 1:** Technical information about tested CPR feedback devices.

Name Manufacturer Country of origin	Audio-visual feedback functions	Technical description
SimCPR®Pro Trainer SIMCPR Medical BV Netherlands	Correct hand position: no Metronome: no Chest compression depth: yes Chest compression frequency: yes Correct chest recoil: no	SimCPR®ProTrainer is a wristband with an integrated acceleration sensor that records acceleration during resuscitation. Audi-visual feedback is transmitted and displayed at the SimCPR®ProTrainer App via Bluetooth.
Relay Response™ App XiMio Health, Inc. United States of America	Correct hand position: no Metronome: yes Chest compression depth: yes Chest compression frequency: yes Correct chest recoil: no	RelayResponse™ is a feedback App that runs on a smartwatch. The smartwatch sensors determine chest compression frequency and chest compression depth. Audi-visual feedback is displayed as numerical feedback.
CorPatchTrainer® SmartResQ ApS Denmark	Correct hand position: no Metronome: yes Chest compression depth: yes Chest compression frequency: yes Correct chest recoil: yes	CorPatchTrainer® is an external device that is placed under the hand during resuscitation. By means of an acceleration sensor, audio-visual feedback is displayed in the CorPatchTrainer® App.
CPRBAND AIO Training CREDO Co., LtD. Korea	Correct hand position: no Metronome: yes Chest compression depth: yes Chest compression frequency: yes Correct chest recoil: no	CPRBAND AIO Training App is connected to the CPRBAND for Training (wristband). Audio-visual feedback is displayed in the App.

### Outcomes

The primary outcome of the study is to measure if CPR metrics (chest compression depth 5–6 cm, chest compression rate 100–120/min, correct hand position, correct recoil) are achieved with different App-linked CPR feedback-devices and how quality was improved compared to controls. Data were collected pseudonymised. Guideline-compliant quality data (ERC guidelines) (chest compression depth (CD), chest compression rate (CR), correct hand position, correct recoil (%)) were measured based on data collected by the manikin. Technical preparation time was defined as time between handover of the device to the participants (start time) until the participants perform the first chest compression (end time). The technical preparation time was measured with a stopwatch by the study assistance. Secondary outcomes are differences in CPR metrics between tested devices and the feedback devices’ impact on participants’ self-confidence in performing CPR. Age, sex, height, weight, previous participation in a first-aid-course, and self-confidence were assessed by means of questionnaire. Self-confidence was measured by asking how confident participants feel (i) “…in having enough theoretical knowledge to resuscitate a OHCA victim”, (ii) “…in having enough practical skills to call the emergency medical service”, (iii) “…in having enough practical skills to check victim's breathing”, (iv) “…in having enough practical skills to perform chest compression”, and (v) “…in having enough practical skills to perform mouth-to-mouth-breathing” on a five-point Likert scale before and after intervention (Mean ± SD) ranging from “not confident” (1 point) to “very confident” (5 points).

### Sample size

The required number of participants was calculated in advance using a statistical power calculation.

The sample size was calculated building upon the study of Metelmann et al.^12^ With a power of 95% and an assumed significance level of 0.05 using a one-sided analysis of variance, a total of 37 participants was calculated. Due to possible drop-out (10%), the sample size was set at 40.

### Randomisation

All participants tested all four App-linked CPR feedback-devices in randomised order. To rule out possible learning or fatigue effect – which are known to set in over long CPR duration^14^ – the order of devices were randomly assigned into four groups (Group 1: ABCD, Group 2: BCDA, Group 3: CDAB, and Group 4: DABC). Randomisation of participants to one of the groups was performed using opaque envelopes.

### Statistical methods

Data analysis was performed with IBM SPSS Statistics 29.0 (SPSS Inc., Chicago, USA). Normally distributed data are described with mean ± SD and compared by using students’ *t*-test. Non-normally distributed data are provided as median and interquartile range (IQR) and compared by using Mann-Whitney-U-test. Wilcoxon-test was performed for comparison of CPR metrics achieved with feedback devices’ use and baseline data. For inter-device comparison, ANOVA was calculated. Statistical significance was accepted as a P value 0.05 or less.

## Results

Forty-seven laypersons were recruited for study participation. Data of forty participants (*n* = 23, 57.5% females; *n* = 17, 42.5% males) with a median age of 22.50 [interquartile range: 21.00–26.00; Min.:19/Max.:30] years were eligible to be analysed in this study. In seven cases, due to technical problems, no data was recorded and participants had to be excluded from statistical analysis. Mean Body-Mass-Index was Mean ± SD: 22.19 ± 3.26(kg * m^−2^). Eighty-five percent (*n* = 34) of participants reported a previous participation in a first-aid course.

### Chest compression depth

SimCPR®ProTrainer was the only device, which resulted in guideline-compliant chest compressions (recommended: 5–6 cm) (Mean ± SD: 5.37 ± 0.76 cm) with improved chest compression depth (*p* < 0.001), and percentage of correct chest compression depth (*p* < 0.001) compared to baseline (Table 2). Percentage of chest compressions with guideline-compliant depth differ between tested devices (*p* = 0.03) and was highest with SimCPR®ProTrainer (Mean ± SD: 79.32 ± 34.41 cm vs. CorPatch® Trainer: Mean ± SD: 57.73 ± 33.44; CPRBAND AIO Training: Mean ± SD: 62.75 ± 35.06; and Relay Response™: Mean ± SD: 60.10 ± 38.39).

**Table 2:** Overall results of CPR metrics in baseline and device groups; CPR = Cardiopulmonary resuscitation; SD = standard deviation.

CPR metrics	No technical support (baseline) Mean ± SD	CorPatch Trainer® Mean ± SD	CPRBand AOI Training Mean ± SD	SimPro®Trainer Mean ± SD	Relay Response™ Mean ± SD	*p-*value^1^
Chest compression depth (cm)	4.96 ± 0.89	4.84 ± 0.72	4.94 ± 0.88	5.37 ± 0.76*	4.95 ± 0.85	***0.02***
Percentage of correct chest compression depth (%)	57.25 ± 42.96	57.73 ± 33.44	62.75 ± 35.06	79.32 ± 34.41*	60.10 ± 38.39	***0.03***
Chest compression rate (compressions/min)	114.03 ± 19.27	106.50 ± 7.72**	110.65 ± 10.13*	117.68 ± 7.88	113.73 ± 8.63	***<0.001***
Percentage of minutes with correct chest compression rate (%)	44.15 ± 43.59	77.25 ± 25.01*	78.07 ± 30.84*	62.13 ± 31.42	77.87 ± 30.05*	***0.04***
Correct hand position (%)	88.85 ± 27.25	92.60 ± 23.64	84.20 ± 33.47	86.70 ± 29.77	88.03 ± 28.39	*0.62*
Percentage of correct chest recoil (%)	49.00 ± 42.20	72.25 ± 24.89**	37.03 ± 39.90**	39.88 ± 36.50†	36.88 ± 37.73†	***<0.001***
Technical preparation time (seconds)	/	27.93 ± 12.52	23.73 ± 10.01	52.25 ± 12.34	14.30 ± 16.25	***<0.001***

1 ANOVA-testing between all devices.

* *p* < 0.001; Wilcoxon-test between device and baseline.

** *p* < 0.01; Wilcoxon-test between device and baseline.

† *p* < 0.05; Wilcoxon-test between device and baseline.

### Chest compression rate

Mean chest compression rate (recommended 100–120 compressions/min) was within guideline-recommendations at baseline (Mean ± SD: 114.03 ± 19.27 per min) and with all devices, highest with SimCPR®ProTrainer (Mean ± SD: 117.68 ± 7.88 per min) vs. CorPatch® Trainer: Mean ± SD: 106.50 ± 7.72 per min; CPRBAND AIO Training: Mean ± SD: 110.65 ± 10.13 per min; and Relay Response™: Mean ± SD: 113.73 ± 8.63 per min.; *p* < 0.001). Frequency decreased when using CorPatch® Trainer Mean ± SD: 106.50 ± 7.72; *p* = 0.008, and CPRBand AOI Training Mean ± SD: 110.65 ± 10.13; *p* < 0.001).

Three out of four devices (CorPatch® Trainer: Mean ± SD: 77.25 ± 25.01%; CPRBAND AIO Training: Mean ± SD: 78.07 ± 30.84%; Relay Response™: Mean ± SD: 77.87 ± 30.05%) supported improved percentage of guideline-conform chest compression rate compared to baseline (Mean ± SD: 44.15 ± 43.59%; all *p* < 0.001). Devices differ in the extent to whether they support participants to achieve a high rate of correct chest compression rate in the 2-minute CPR setting (*p* = 0.04). CPRBAND AOI Training supported the highest percentage of correct chest compression rate.

### Correct hand position

None of the tested devices improved the percentage of correct hand position during CPR in layperson compared to baseline. Moreover, devices showed no differences between achieved percentage of correct hand position (*p* = 0.62).

### Chest recoil

CorPatch® Trainer as the only device with audio-visual chest recoil instructions showed improved chest recoil rates in layperson CPR (Mean ± SD: 72.25 ± 24.89%) compared to baseline (Mean ± SD: 49.00 ± 42.20%; *p* < 0.01). All other devices resulted in significantly lower chest recoil rates compared to unassisted CPR (CPRBAND AIO Training: Mean ± SD: 37.03 ± 39.90%; *p* < 0.01, SimCPR®ProTrainer: Mean ± SD: 39.88 ± 36.50%; *p* = 0.03; Relay Response™: Mean ± SD: 36.88 ± 37.73%; *p* = 0.02).

### Technical preparation time

Feedback-devices differ in the duration until technical preparation was finished by the participants (*p* < 0.001) and was highest with SimCPR®ProTrainer Mean ± SD: 52.25 ± 12.34 seconds and lowest with Relay Response™: Mean ± SD: 14.30 ± 16.25 seconds (vs. CorPatch® Trainer: Mean ± SD: 27.93 ± 12.52 seconds; CPRBAND AIO Training: Mean ± SD: 23.73 ± 10.01 seconds).

### Self-confidence in own CPR performance

Participant’s self-confidence in performing CPR increased significantly after using feedback devices (before: Mean ± SD: 3.30 ± 0.82 vs. after: Mean ± SD: 3.86 ± 0.60; Cohen’s *d*: 1.09; *p* < 0.001).

## Discussion

This is the first study to compare CPR quality results achieved when using the different App-linked CPR feedback-devices for layperson that are currently available on the market. Three major findings can be assumed: (1) None of the tested CPR feedback devices obtained overall guideline-compliant CPR, (2) CPR quality when using feedback-devices differs significantly, and adequate chest compression depth (5–6 cm) was only reached with one out of four studied App-linked CPR real-time feedback-devices (SimCPR®ProTrainer). (3) If feedback-devices integrate audio-visual instructions about guideline-compliant chest recoil, CPR performance of laypeople was further improved (CorPatch® Trainer), but decreases with all other devices. Also, Tanaka et al. found improved chest recoil rates in laypeople when audio-visual feedback about chest recoil was giving.^15^ If concentrating on the feedback distracts participants from paying attention to the other non-feedback parameters, it becomes of high importance that feedback-devices offer feedback on all CPR metrics to prevent low-quality CPR in laypeople.

In line with our study results, previous studies examining apps and feedback-devices for layperson’ use show that not all CPR metrics could be reached guideline-compliant.^8^, ^12^, ^16^, ^17^, ^18^ In particular, the correct compression depth, which is one of the central CPR metrics in resuscitation,^1^, ^3^ was only correctly supported by one device in our study. Also, other studies demonstrate that the guideline-compliant chest compression depth is not achieved with resuscitation apps or CPR feedback-devices.^8^, ^19^

Smartwatch-Apps were found to be more adequate in measured quality and offer lower error susceptibility than smartphone applications.^8^, ^20^, ^21^ The difficulty with smartphone Apps is to hold/operate the smartphone and perform resuscitation at the same time.^8^ In contrast to telephone-assisted CPR, our study demonstrated that the use of App-linked feedback-devices requires time for preparation (i.e., opening smartphone application, fastening of the device on the wrist, and/or starting the external device and placing it on the chest). Also Metelmann et al. found higher hands-off time when App-assisted CPR was performed compared to CPR without technical support.^22^ As demonstrated in our study, technical preparation time differs strongly and should be critically discussed when feedback devices should be used in real-case scenarios.

Lacking self-confidence in performing CPR is an important obstacle for initiating bystander activity in real-life cardiac arrest situation.^23^ As shown in other studies, CPR trainings are an effective method to improved self-confidence in laypersons.^24^, ^25^ It can be assumed that training CPR with App-linked feedback devices, as shown in our study, increases laypeople's confidence in CPR. Although, 85% of study participants report that they have visited a first-aid course beforehand, self-confidence in CPR performance was further improved by using CPR feedback devices. To counteract this, feedback devices can serve as a refresher and can be used during or after visiting CPR or first-aid courses.^22^ Since there is currently discussion about low-cost training options (e.g. PET bottles as a substitute for real resuscitation manikins), feedback devices are interesting supplementary products with which low-cost resuscitation settings can also be realised if they are further validated.^26^, ^27^

### Future demands and quality improvement

As demonstrated with our study and confirmed in current literature, quality of CPR performance with feedback-devices’ support is lacking in all CPR metrics and evidence for usefulness merits are discussing.^8^ Due to a steadily increasing number of people worldwide using apps and App-related technologies,^8^, ^9^ CPR feedback-devices offer an effective and low-cost possibility to raise up CPR competences in societies. It can be assumed that the number of App-linked CPR feedback-devices will increase in future.^28^ There is a strong need for improved functionality, validation, and user friendliness^8^, ^12^, and current devices should be improved, accordingly. Systematic quality control, as recommended by Metelmann et al., could ensure that apps and devices provide guideline-compliant content and sufficient usability.^12^ It can be discussed that it would most probably make sense that these devices were improved by implementing more medical expert involvement and evidence-based content.^29^ This can be ensured by defining quality criteria based on quality principles for mobile-health-applications, that have been published by the Association of the Scientific Medical Societies.^30^

## Limitations

As this study is a simulation trial in a standardised study setting using manikins, some limitations in the interpretation of our results need to be considered. In our study, CPR duration was limited to two minutes to allow standardised comparison of CPR performance. Users may achieve a better level of CPR training in real-life if they train longer with the device. Moreover, in real-case scenarios, CPR duration is often required to be 6–8 minutes and longer until the Emergency Medical Services arrive on scene. A positive influence on reduced fatigue when using technical feedback during CPR has been shown previously.^14^ Although our study design is in line with other studies,^22^, ^31^ measured CPR metrics could be affected both, positively or negatively. All participants received standardised introductions in using the App-linked feedback-device, which might not reflect home-use of these technologies by layperson. In particular, it must be assumed that the technical preparation time will be even longer if layperson have to familiarise themselves with the technology at home instead of the standardised instructions. The duration and intensity of previous BLS instructions may have an influence on the CPR quality that layperson reach with CPR feedback devices. For this reason, the study results may not be applicable to all training settings.

## Conclusion

Although, CPR feedback devices show improved CPR performance for layperson in some metrics, none of the tested CPR feedback devices supported layperson in overall adequate CPR performance. More and better technical functionality is necessary to fully utilise the potential of CPR feedback devices and to prevent a worsening of CPR performance when layperson use this technology.

## CRediT authorship contribution statement

**Sabine Wingen:** Writing – original draft, Visualization, Validation, Supervision, Resources, Project administration, Methodology, Investigation, Formal analysis, Data curation, Conceptualization. **Nele Großfeld:** Writing – original draft, Visualization, Validation, Formal analysis, Data curation, Conceptualization. **Niels-Benjamin Adams:** Writing – review & editing, Visualization, Methodology, Data curation, Conceptualization. **Antonia Streit:** Writing – review & editing, Conceptualization. **Jan Stock:** Writing – review & editing, Resources, Investigation, Conceptualization. **Bernd W. Böttiger:** Writing – review & editing, Resources. **Wolfgang A. Wetsch:** Writing – original draft, Validation, Supervision, Methodology, Investigation, Formal analysis, Data curation, Conceptualization.

## Declaration of competing interest

The authors declare the following financial interests/personal relationships which may be considered as potential competing interests: ‘Sabine Wingen is executive personal assistant of the executive board of the German Resuscitation Council (GRC) and receive fees for lectures and consulting from FOM Hochschule für Oekonomie & Management and L2R GmbH. Nele Großfeld works for the L2R GmbH. Jan Stock is Head of the L2R GmbH and Project Manager at the Hans Peter Esser GmbH. Bernd W. Böttiger is treasurer of the European Resuscitation Council (ERC), Founder of the ERC Research NET, Chairman of the German Resuscitation Council (GRC), Member of the „Advanced Life Support (ALS) Task Force of the International Liaison Committee on Resuscitation (ILCOR), Member of the Executive Committee of the German Interdisciplinary Association for Intensive Care and Emergency Medicine (DIVI), Founder of the “Deutsche Stiftung Wiederbelebung”, Federal Medical Advisor of the German Red Cross (DRK), Member of the Advisory Board of the “Deutsche Herzstiftung”, Co-Editor of “Resuscitation”, Editor of the Journal “Notfall + Rettungsmedizin”, Co-Editor of the Brazilian Journal of Anesthesiology. He received fees for lectures from the following companies: Forum für medizinische Fortbildung (FomF), ZOLL Medical Deutschland GmbH, C.R. Bard GmbH, Becton Dickinson GmbH. Wolfgang A. Wetsch, Niels-Benjamin Adams, and Antonia Streit have no conflicts of interest.’.
